# ZFP36 protects lungs from intestinal I/R-induced injury and fibrosis through the CREBBP/p53/p21/Bax pathway

**DOI:** 10.1038/s41419-021-03950-y

**Published:** 2021-07-08

**Authors:** Yongmei Cao, Weifeng Huang, Fang Wu, Jiawei Shang, Feng Ping, Wei Wang, Yingchuan Li, Xuan Zhao, Xiaoping Zhang

**Affiliations:** 1 Department of Critical Care Medicine, Shanghai Jiaotong University Affiliated Sixth People’s Hospital, No. 600, Yishan Rd, Xuhui District, Shanghai, 201499 China; 2 Department of Anesthesiology, Shanghai Tongji University Affiliated Tenth People’s Hospital, No. 301, Middle Yanchang Road, Shanghai, 200072 China; 3 Department of Interventional Vascular, Shanghai Tenth People’s Hospital, Tongji University, Shanghai, 200072 China; 4 Shanghai Center of Thyroid Diseases, Tongji University School of Medicine, Shanghai, 200072 China; 5 Zhuhai Precision Medical Center, Zhuhai People’s Hospital, Zhuhai Hospital Affiliated with Jinan University, Jinan University, Zhuhai, Guangdong 519000 P.R. China

**Keywords:** Transcription, Respiratory tract diseases

## Abstract

Acute lung injury induced by ischemia–reperfusion (I/R)-associated pulmonary inflammation is associated with high rates of morbidity. Despite advances in the clinical management of lung disease, molecular therapeutic options for I/R-associated lung injury are limited. Zinc finger protein 36 (ZFP36) is an AU-rich element-binding protein that is known to suppress the inflammatory response. A ZFP36 binding site occurs in the 3ʹ UTR of the cAMP‐response element-binding protein (CREB) binding protein (CREBBP) gene, which is known to interact with apoptotic proteins to promote apoptosis. In this study, we investigate the involvement of ZFP36 and CREBBP on I/R-induced lung injury in vivo and in vitro. Intestinal ischemia/reperfusion (I/R) activates inflammatory responses, resulting in injury to different organs including the lung. Lung tissues from ZFP36-knockdown mice and mouse lung epithelial (MLE)-2 cells were subjected to either Intestinal I/R or hypoxia/reperfusion, respectively, and then analyzed by Western blotting, immunohistochemistry, and real-time PCR. Silico analyses, pull down and RIP assays were used to analyze the relationship between ZFP36 and CREBBP. ZFP36 deficiency upregulated CREBBP, enhanced I/R-induced lung injury, apoptosis, and inflammation, and increased I/R-induced lung fibrosis. In silico analyses indicated that ZFP36 was a strong negative regulator of CREBBP mRNA stability. Results of pull down and RIP assays confirmed that ZFP36 direct interacted with CREBBP mRNA. Our results indicated that ZFP36 can mediate the level of inflammation-associated lung damage following I/R via interactions with the CREBBP/p53/p21/Bax pathway. The downregulation of ZFP36 increased the level of fibrosis.

## Introduction

Acute lung injury, resulting from systemic inflammatory responses as a consequence of ischemia–reperfusion (I/R), has limited therapeutic options and is associated with high morbidity [1, 2]. Injury to the lung from I/R can occur through a global response from thoracic surgery to other organs, such as the liver [3], heart [4], kidneys [5], and intestine [6]. Intestinal ischemia–reperfusion (I/R) injury develops when the blood fow to the intestines decreases, followed by the reestablishment of the blood supply to the ischemic tissue. Intestinal I/R injury results in intestinal mucosal barrier dysfunction, which may cause severe local and systemic infammation [6]. The inflammation triggered by I/R involves many proinflammatory factors including neutrophils and cytokines [7]. Neutrophils that are recruited to the lungs following I/R produce an excessive amount of free radicals and reactive oxygen species (ROS) to cause further injury and necrosis [8].

Although the inflammatory process in acute lung injury is well-studied, less is known about the role played by apoptosis and fibrosis. However, drugs that ameliorate the inflammatory processes associated with I/R can reduce levels of fibrosis in the lung and alleviate injury [9]. Elevated levels of transforming growth factor (TGF)-β1 and tumor necrosis factor (TNF)-α in the lung are associated with increased levels of fibrosis [10]. However, the role of TGF-β1 in fibrosis is contradictory. Although TGF-β1 is thought to reduce fibrosis by regulating neutrophil apoptosis through IL-6 [11], it is also believed to promote fibrosis and apoptosis through Egr-1 [12, 13]. Overexpression of TNF-α following induction by inflammatory factors is a well-documented characteristic of fibrosis [14, 15]. TNF-α is mainly derived from macrophages and accumulates rapidly in response to injury [16, 17]. In conjunction with interleukins (ILs), TNF-α is known to activate the NF-κB and JAK/STAT pathway in response to lung injury to induce the expression of several major chemokines including CXCL13, which is involved in pulmonary fibrosis [18].

Zinc finger protein 36 (ZFP36, also known as tristetraprolin) is an AU-rich element-binding protein that suppresses the inflammatory response [19]. ZFP36 promotes mRNA decay by binding to mRNA 3ʹUTR and is known to target TNF-α [20]. Mice that are deficient in ZFP36 have high levels of TNF-α and are known to develop severe inflammatory syndrome as a consequence [21]. Moreover, overexpressing ZFP36 in mouse embryonic fibroblasts inhibits the induction of p65/NF-κB by cAMP‐response element-binding protein (CREB) binding protein (CREBBP) [22]. CREB and CREBBP are associated with the regulation of several proteins involved in apoptosis, such as BCL2 and Bax [23, 24]. The inhibition of CREBBP is known to protect against apoptotic injury during I/R, and it has been proposed that the binding of CREBBP to CREB triggers apoptosis [25].

Thus, there is no research about the relationship between ZFP36 and CREBBP in I/R. In this study, we investigate the involvement of ZFP36 and CREBBP in I/R-induced lung injury and the interaction of ZFP36 with CREBBP mRNA 3ʹUTR in mouse lung epithelial (MLE) cells and a murine model of I/R.

## Results

### ZFP36 reduces the severity of intestinal I/R-induced acute lung injury

To study the involvement of ZFP36 in I/R-induced acute lung injury we first created an intestine ischemia model with reperfusion in C57BL/6 mice. The injury to lung tissue, represented by arterial blood partial pressure of oxygen (PaO_2_), lung water content, and bronchoalveolar lavage fluid (BALF) protein content, increased significantly with reperfusion and then stabilized after 60 min (Fig. S1A–C). Therefore, 60 min of intestine ischemia followed by 60 min of reperfusion was selected as the best representation of lung damage in the mouse model. After 60 min of reperfusion, hematoxylin and eosin (H&E)-stained tissue and measurements of IL-1β, TNF-α, and IL-6 levels indicated significant lung damage with alveolar edema and inflammatory cellular sequestration (Fig. S1D–G).

**Fig. 1:**
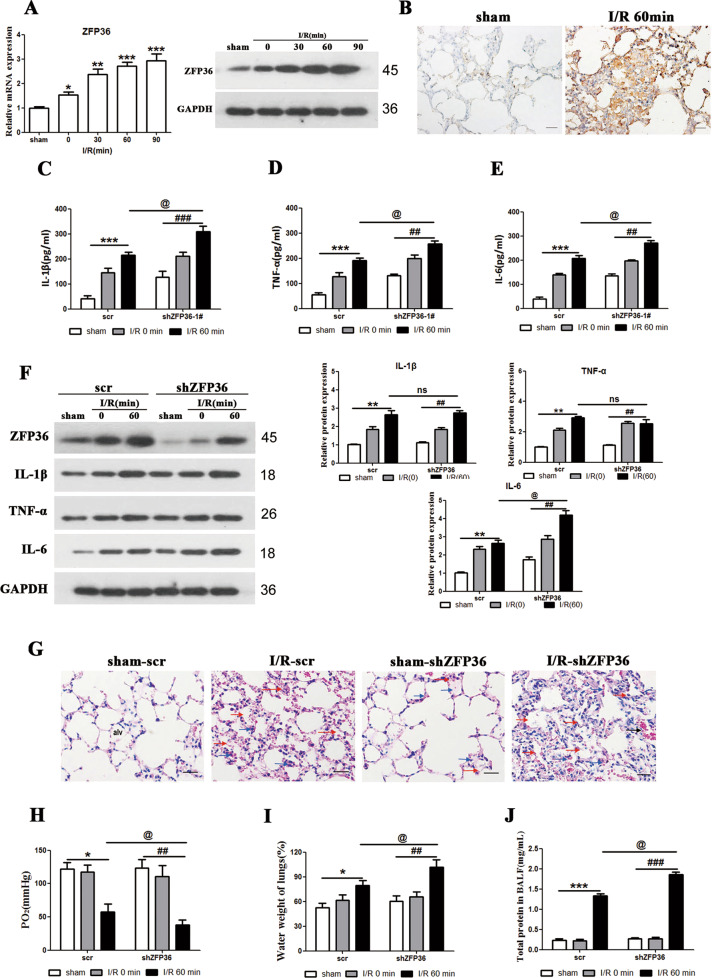
Role of ZFP36 in intestinal ischemia–reperfusion (I/R)-induced acute lung injury. C57BL/6 mice were subjected to 60 min of intestinal ischemia followed by 0, 30, 60, and 90 min of reperfusion as indicated. Sham mice were included as the control. **A** ZFP36 mRNA and protein expression in lung tissues were analyzed by RT-qPCR and Western blot (*n* = 6 per group, **P < 0.01). **B** Immunohistochemical staining of lung tissues for ZFP36 of sham and I/R 60 min. Scale bars: 50 μm. **C**–**F** Levels of IL-1β (**C**), TNF-α (**D**), and IL-6 (**E**) were measured by ELISA and Western blotting (**F**). **G** H&E staining of lung tissues. Red arrows outline collapsed alveoli, blue arrows outline multiple inflammatory cells infilitration, black arrows outline bronchial hemorrhage. Scale bars: 50 μm. **H**–**J** Arterial blood PaO_2_ (**H**), lung water content (**I**), and BALF protein content (**J**) were measured.

We used the intestinal I/R-induced acute lung injury in mice to assess the levels of ZFP36 in lung tissue. ZFP36 mRNA and protein expression gradually increased compared with the sham group as the length of reperfusion increased (*p* < 0.05, *p* < 0.01, *p* < 0.001) (Fig. 1A). High levels of ZFP36 were detected by immunohistochemical staining in the lung tissues of the I/R-induced mouse model compared with sham-operated mice (Fig. 1B). When the expression of ZFP36 is inhibited by RNA interference, the protein levels of IL-1β, TNF-α, and IL-6 increase significantly, indicating that the inflammatory response is elevated when ZFP36 is suppressed (Fig. 1C–F). This is also demonstrated in H&E-stained lung sections, with increased alveolar edema in tissue when the expression of ZFP36 is suppressed (Fig. 1G). Arterial blood PaO_2_ was lowered on suppression of ZFP36 (*p* < 0.05, *p* < 0.01), whereas lung water content and the protein content of BALF was significantly increased in shZFP36 group (*p* < 0.05, *p* < 0.01, *p* < 0.001) (Fig. 1H–J). This indicates that the severity of lung injury is increased when ZFP36 is suppressed leading to reduced availability of blood oxygen and increased inflammation. Overall, our results suggest that ZFP36 is upregulated during the process of lung injury and suppresses inflammatory responses by mediating the regulation of inflammatory related genes.

### ZFP36 interacts with CREBBP mRNA to prevent the promotion of intestinal I/R-induced acute lung injury

**Fig. 2:**
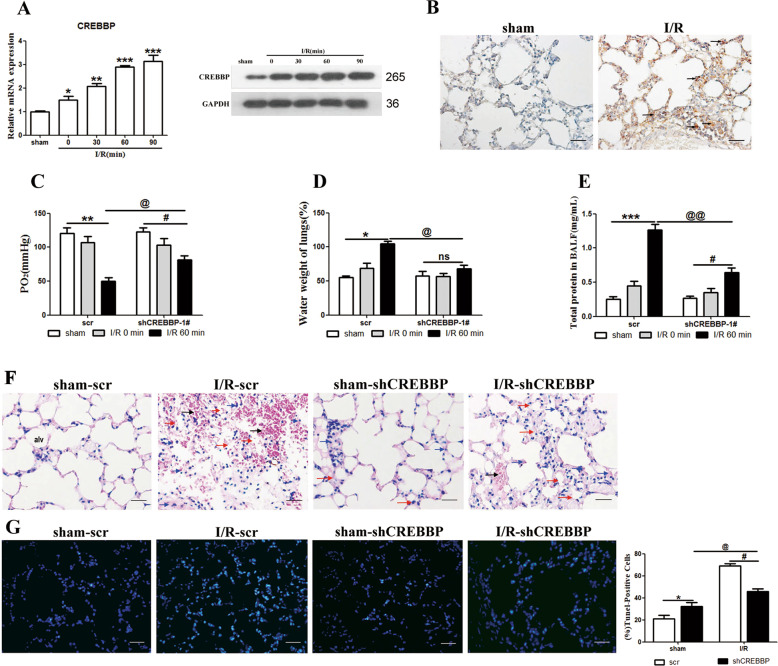
Role of CREBBP in intestinal ischemia–reperfusion (I/R)-induced acute lung injury. **A** CREBBP mRNA and protein expression in lung tissues were analyzed by RT-qPCR and Western blotting (*n* = 6 per group, **P < 0.01). **B** Immunohistochemical stain of lung tissues for CREBBP of sham and I/R 60 min group. Scale bars: 50 μm. **C**–**E** Arterial blood PaO_2_ (**C**), lung water content (**D**), and BALF protein content (**E**) were measured. **F** Representative H&E staining of lung sections. Scale bars: 50 μm. **G** TUNEL assay was performed on lung sections from each group and TUNEL-positive cells were determined.

Next, we assessed the involvement of CREBBP in the intestinal I/R-induced acute lung injury model. CREBBP mRNA expression and protein levels were significantly increased in the lung tissue of the intestinal I/R-induced model (*p* < 0.05, *p* < 0.01, *p* < 0.001) (Fig. 2A) and elevated levels of CREBBP were found in I/R lung tissue sections (Fig. 2B). The inhibition of CREBBP by RNA interference resulted in the increased availability of oxygen in arterial blood (*p* < 0.05, *p* < 0.01) and reduced level of lung injury as indicated by lung water content and BALF protein content (*p* < 0.05, *p* < 0.01, *p* < 0.001) (Fig. 2C–E). Furthermore, CREBBP was associated with an increased level of cell death in I/R injured tissue as demonstrated in H&E lung tissue sections and by TUNEL-positive cells (*p* < 0.05) (Fig. 2F, G). These results indicate that CREBBP could be involved in the apoptosis of cells in the lungs following I/R acute lung injury.

**Fig. 3:**
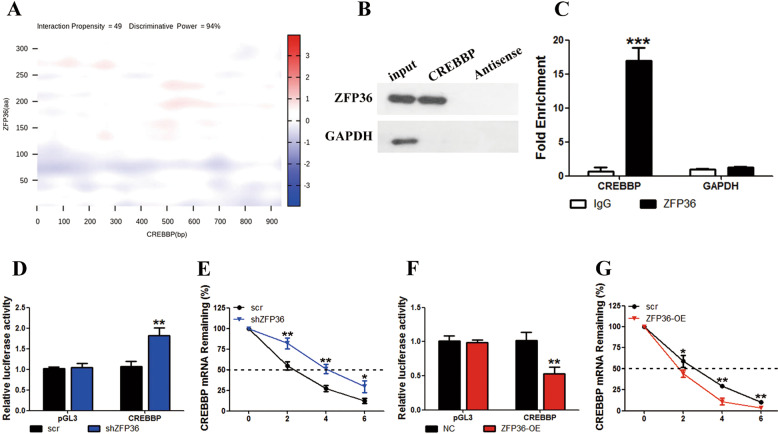
ZFP36 destabilizes CREBBP mRNA by binding to it. **A** catRAPID fragment-based prediction assays of interaction between ZFP36 and CREBBP identified a strong interaction with high confidence levels (interaction propensity = 49 and discriminative power = 94%). **B** Western blotting analysis followed by RNA pull-downs, incubated with lysed lung epithelial-2 cell supernatant. GAPDH was used as a control. **C** Fold enrichment of CREBBP expression in RIP assays performed with ZFP36-coated beads. **D**, **F** Lung epithelial-2 cells underexpressing (**D**) or overexpressing (**F**) ZFP36 were stably transfected with luciferase constructs carrying the 3ʹUTR of the gene encoding CREBBP or empty vector (pGL3). Luciferase activities were measured and normalized to the activities obtained in pGL3-transfected cells (*n* = 3 in every group, **P < 0.01). **E**, **G** Remaining CREBBP mRNA levels were measured in lung epithelial-2 cells by real-time PCR (*n* = 3 in every group).

We used a catRAPID fragment-based prediction assay to assess whether ZFP36 interacts with the 3ʹUTR of CREBBP. A strong interaction with high confidence levels was identified in the binding assay (interaction propensity, 49; discriminative power, 94%) (Fig. 3A). Western blot analysis of RNA pull down and an immunoprecipitation assay with ZFP36-coated beads confirmed the ZFP36/CREBBP interaction in MLE-2 cells (*p* < 0.001) (Fig. 3B, C). We also performed a luciferase assay with the 3ʹUTR of CREBBP or ZFP36 either increased or decreased expressed in MLE-2 cells. The underexpression of ZFP36 resulted in greater luciferase activity associated with the mRNA 3ʹUTR of the gene encoding CREBBP whereas overexpression reduced luciferase activity (*p* < 0.01) (Fig. 3D, F). Moreover, the remaining time of CREBBP mRNA was higher in MLE-2 cells when ZFP36 was underexpressed compared to when ZFP36 was overexpressed (*p* < 0.05, *p* < 0.01) (Fig. 3E, G), which indicates that ZFP36 could promote the decay of CREBBP mRNA by binding to it.

### Effect of ZFP36 silencing on acute lung injury induced inflammation and apoptosis

**Fig. 4:**
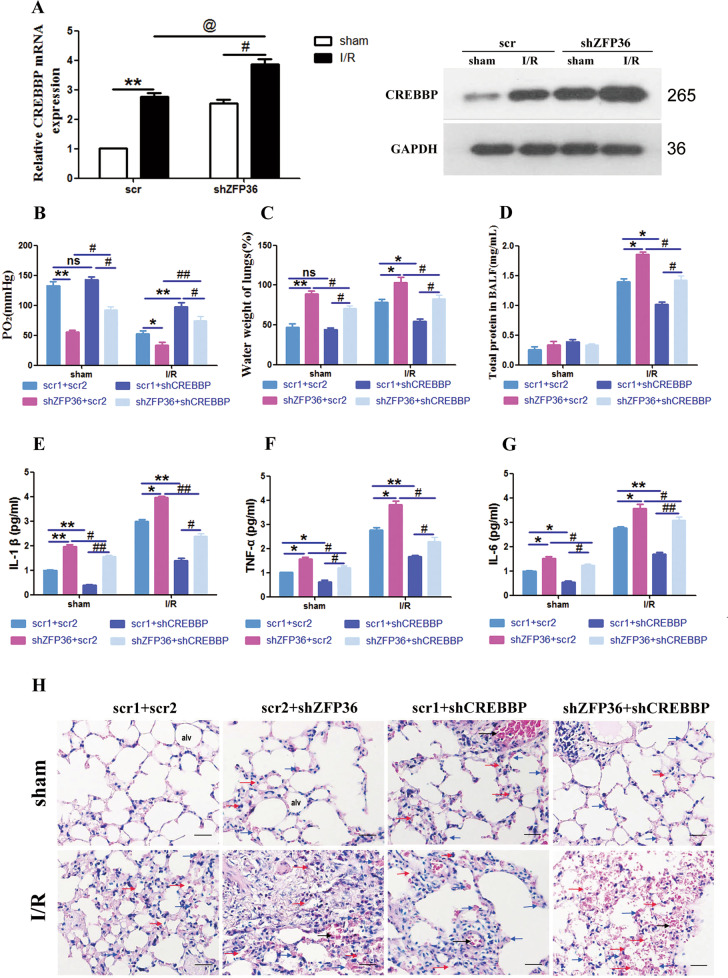
Effect of ZFP36 and CREBBP deficiency on ischemia–reperfusion (I/R)-induced lung damage and inflammation in vivo. **A** CREBBP mRNA and protein expression in lung tissues of mice transfected with shZFP36 or empty vector (scr) were analyzed by RT-qPCR (*n* = 6 per group, **P < 0.01) and Western blot. **B**–**D** Arterial blood PaO_2_ (**B**), lung water content (**C**), and the protein content of BALF (**D**) were measured. **E**–**G** Levels of IL-1β (**E**), TNF-α (**F**), and IL-6 (**G**) were measured by ELISA. **H** H&E staining of lung sections, scale bar: 50 μm.

To confirm our finding that ZFP36 could prevent CREBBP-induced lung injury through mRNA degradation, we measured the effect of altering the expression of ZFP36 on the level of CREBBP mRNA in the intestinal I/R-induced lung injury mouse model. CREBBP mRNA expression and protein levels were significantly increased in the lung tissue of the I/R model when ZFP36 expression was knocked down (*p* < 0.05) (Fig. 4A). The severity of lung injury (measured by arterial blood PaO_2_, lung water content, and BALF protein content) (*p* < 0.05, *p* < 0.01) (Fig. 4B–D) and inflammatory response (measured by levels of IL-1β, TNF-α, and IL-6 levels) were highest in the lung tissue of the I/R model with ZFP36 knockdown (Fig. 4B–G). In contrast, downregulating the expression of CREBBP by RNA interference significantly reduced the severity of lung injury. H&E-stained lung sections of the mice with I/R confirmed that alveolar edema was the most pronounced in the absence of ZFP36 (Fig. 4H).

**Fig. 5:**
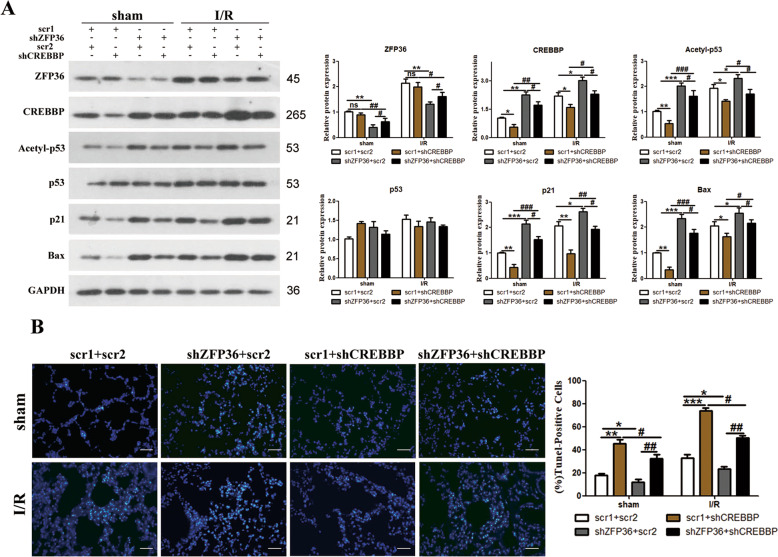
ZFP36 inhibits ischemia–reperfusion (I/R)-induced apoptosis via the CREBBP/p53/p21/Bax pathway. **A** Expression levels of apoptosis-related protein were analyzed by Western blotting. **B** TUNEL assay was performed on lung sections from each group and TUNEL-positive cells were determined.

To reaffirm our findings, we repeated the experiment in MLE-2 cells subjected to hypoxia followed by regeneration (H/R) and further investigated the expression of apoptosis-related proteins and cell viability (Fig. S2). When the expression of ZFP36 is downregulated, the protein levels of CREBBP, Bax, p21, and acetyl-p53 are increased and cell viability is reduced, indicating that ZFP36 inhibits apoptosis via the CREBBP/p53/p21/Bax pathway in vitro. We repeated the experiment in the I/R mouse model with the expression of ZFP36 or CREBBP downregulated (Fig. 5). The expression of apoptosis-related proteins and TUNEL assays performed on lung sections from the mice demonstrated that ZFP36 inhibits I/R-induced apoptosis through the CREBBP/p53/p21/Bax pathway (*p* < 0.05, *p* < 0.01, *p* < 0.001) (Fig. 5B).

### ZFP36 protects against intestinal I/R-induced lung fibrosis by inhibiting epithelial–mesenchymal transition (EMT)

**Fig. 6:**
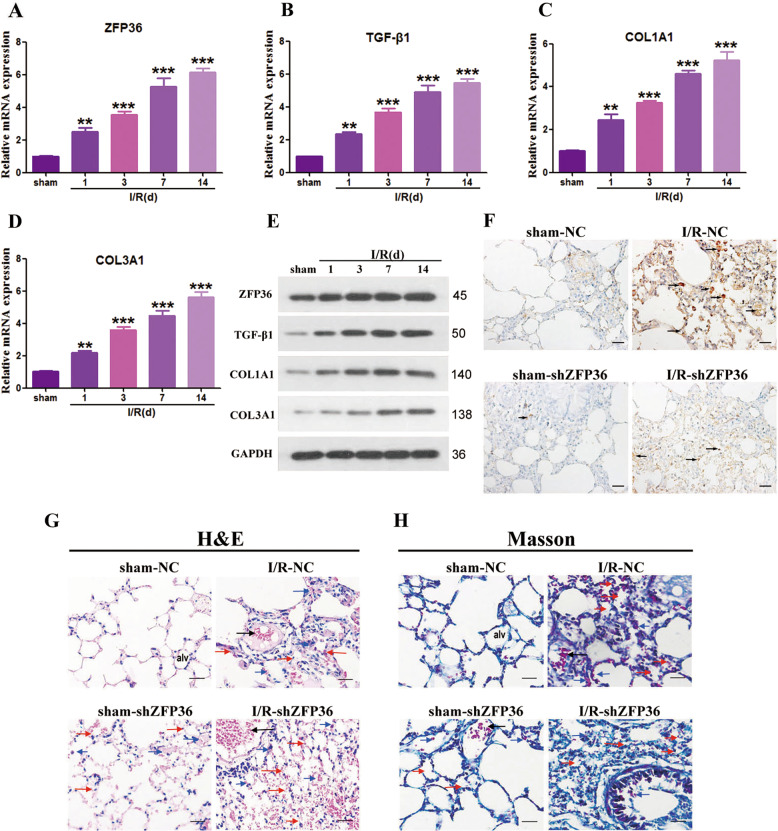
Role of ZFP36 in intestinal ischemia–reperfusion (I/R)-induced lung fibrosis. C57BL/6 mice were subjected to 60 min intestine ischemia followed by 60 min reperfusion, and then detections were performed at 1, 3, 7, and 14 days. Sham mice were included as a control. **A**–**D** ZFP36 (**A**), TGF-β1 (**B**), COL1A1 (**C**), and COL3A1 (**D**) mRNA expression in lung tissues of each group were analyzed by RT-qPCR (*n* = 6 per group, **P < 0.01) and Western blot. **E** ZFP36, TGF-β1, COL1A1, and COL3A1 protein expression in lung tissues of each group by Western blotting. **F** Immunohistochemical staining of lung tissues of mice with stable knockdown of ZFP36 or scr for sham and I/R after 14 days. Scale bars: 50 μm. **G**, **H** H&E stain (**G**) and Masson trichrome (**H**)-stained lung sections. Scale bar: 50 μm.

To determine whether ZFP36 could alleviate intestinal I/R-induced lung fibrosis associated lung injury, proteins related to fibrosis (TGF-β1, COL1A1, and COL3A1) were measured in the I/R mouse model 14 days after the I/R procedure (Fig. 6A–E). There was a significant increase in the accumulation of fibrosis-related proteins in the lung tissue of mice 14 days after I/R (*p* < 0.01, *p* < 0.001). The detection of ZFP36 by the immunohistochemical staining of lung tissue in mice with ZFP36 knockdown revealed that high levels of ZFP36 were related to reduced levels of alveolar edema and lung injury (Fig. 6F). Intensely stained collagen fibers surrounding the vessels and bronchioles in the lung tissue in Masson trichrome-stained sections indicated a greater presence of fibrosis in the I/R mice with ZFP36 knockdown (Fig. 6G, H). Experiments in vitro confirmed that the downregulation of ZFP36 increased the level of fibrosis (Fig. S3). Proteins involved in fibrosis and EMT all indicated that the silencing of ZFP36 increased the level of fibrotic activity following I/R-induced injury.

## Discussion

Acute lung injury derived from pulmonary inflammation following I/R has a complex etiology [26, 27]. Presently, there are no markers to predict the severity of lung injury following I/R and there are no molecular-based therapies either [28]. However, some proinflammatory cytokines (e.g., TNF-α, IL-1β, and IL-6) are differentially regulated following I/R and several of these are negatively correlated with the expression of ZFP36 [29]. ZFP36 has been found to suppress inflammatory responses by destabilizing the mRNA of genes encoding proteins associated with inflammation [21, 30]. Therefore, we aimed to investigate the molecular role of ZFP36 behind the development of lung injury in an animal model of I/R.

Our findings that ZFP36 is upregulated in lung injury derived from intestinal I/R, and suppression of ZFP36 led to increased levels of proinflammatory proteins (IL-1β, TNF-α, and IL-6) and severity of lung injury is similar to other studies [31]. In this study, we examined the role of ZFP36 in mRNA degradation by assessing its interactions with CREBBP, which promotes apoptosis following I/R and possesses an AU-rich 3ʹUTR ZFP36 binding site. We confirmed that ZFP36 interacts with the 3ʹUTR binding site in CREBBP. We also found that ZFP36 is negatively correlated with CREBBP. Our results suggest that ZFP36 is upregulated during the process of lung injury and suppresses inflammatory responses by regulating the mRNA stability of CREBBP. We found that ZFP36 can mediate the inflammatory response by destabilizing RNA as reported previously in independent studies [31–33]. Marchese et al. proposed that ZFP36 degrades mRNA through the recruitment of the Ccr4/Caf1/Not deadenylase complex and that ZFP36 is recruited by the activation of p38-MK2 signaling [34].

To determine the pathway involved in the suppression of I/R-induced injury promoted by the ZFP36/CREBBP interaction, we assessed the expression and activation of proteins involved in inflammation-mediated apoptosis. We found that downregulating the expression of ZFP36 could increase the protein levels of CREBBP, Bax, p21, and acetyl-p53 and reduce cell viability. The degradation of CREBBP mRNA by ZFP36 suppresses the expression and activation of apoptotic-related proteins. Therefore, ZFP36 could inhibit apoptosis through the CREBBP/p53/p21/Bax pathway. Similarly, in a rat myocardial I/R injury model, Yang et al. [25] found that downregulating of CREBBP resulted in decreased p53 acetylation activity and levels of Bax and p21. However, the downregulation of CREBBP, in this case, did not occur by the degradation of CREBBP mRNA but by the competitive endogenous binding of microRNA.

Finally, we examined whether ZFP36 could alleviate the fibrosis-related to I/R-induced lung injury by measuring markers associated with fibrosis and EMT in mice with ZFP36 knockdown. Pulmonary fbrosis is a disease induced by lung injury, which may develop as the result of repeated stimuli, with early cycles of injury to alveolar epithelial and endothelial cells, followed by inflammation and attempted repair [35]. The levels of fibrosis and EMT were increased when ZFP36 was knockdown (Fig. S3). Montorsi et al. [30] reported similar results; ZFP36 inhibits the expression of transcription factors that are involved in EMT and is negatively correlated with the Wnt/β-catenin pathway in colorectal cancer. The suppression of EMT by ZFP36 is a recurring pattern in other cancers, such as ovarian cancer, hepatocellular carcinoma, and lung cancer [36–38]. In cancer cells, ZFP36 is believed to act as an EMT suppressor by binding to AU-rich elements in the mRNA 3′UTRs of *Twist1* and *Snail1* [39].

To summarize, ZFP36 suppressed the inflammatory response in I/R-associated pulmonary inflammation through the destabilization of CREBBP mRNA. Analysis of lung tissues in ZFP36-knockdown mice by Western blotting, immunohistochemistry, and real-time PCR showed that ZFP36 deficiency upregulated CREBBP, enhanced I/R-induced lung injury, apoptosis, and inflammation, and increased I/R-induced lung fibrosis. In silico analyses revealed that ZFP36 is a strong negative regulator of CREBBP mRNA stability. Experimental verification of this association revealed a direct interaction between ZFP36 and an AU-rich 3ʹUTR in CREBBP to regulate cell inflammation and apoptosis negatively through the p53/p21/Bax axis in MLE-2 cells.Thus these results of our study indicate that ZFP36 interacts with the mRNA of CREBBP and may be useful as a molecular marker or have a potential role in alleviating inflammation-associated lung damage.

## Materials and methods

### Animal model of intestinal ischemia/reperfusion-induced acute lung injury

Eight-week-old C57BL/6 J mice (purchased from the Chinese Science Academy) were used in accordance with the guidelines specified by the Animal Care and Usage Committee of Tongji University. The animals were fasted with free access to water for 24 h before the I/R procedure. After animals were sedated with an intraperitoneal injection of sodium pentobarbital (50 mg/kg) the superior mesenteric artery was clamped with an atraumatic microvascular clip. After 60 min the clamps were removed, and intestinal reperfusion was established for the specified times. The procedure was replicated in sham-operated mice without vascular clamping.

### Real-time PCR

Total RNA was extracted from tissue using Trizol Reagent (Invitrogen, Carlsbad, CA, USA) following the manufacturer’s protocol. Real-time PCR was performed using an ABI Prism 7,500 system (PE Applied Biosystems, Waltham, MA, USA) and the following conditions: 95 °C for 5 min; 45 cycles of 95 °C for 30 s, 58 °C for 30 s, and 72 °C for 30 s; then a final extension of 72 °C for 1 min. U6 was used as an internal control and the 2^−ΔΔCt^ method was used to calculate the relative expression.

### Western blot analysis

The protein levels of ZFP36, CREBBP, IL-1β, TNF-α, IL-6, TGF-β1, COL1A1, and COL3A1 were determined by Western blot analysis. Protein was first extracted from cells and lung tissue using RIPA buffer containing protease and phosphatase inhibitors (Beyotime, Wuhan, China) for 30 min. Equivalent samples of protein (40 μg) were separated by 10% SDS-polyacrylamide electrophoresis and then transferred to a PVDF membrane (Millipore, Bedford, MA, USA). Membranes were first blocked in 5% non-fat dry milk for 2 h and then incubated overnight at 4 °C with relevant primary antibodies following manufacturers’ recommendation. Finally, membranes were incubated with horseradish peroxidase-conjugated secondary antibody (C13091, Applygen Technologies Inc., China, 1:1000) at room temperature for 1 h and immunoreactive proteins were visualized by using an enhanced chemiluminescence detection kit (Pierce, Rockford, IL, USA). Images of visualized blot bands were acquired and Gapdh was used as the loading control. The primary antibodies are listed as follows: ZFP36 (#71632, Cell Signaling Technology, USA, 1:1000), CREBBP (ab2832, Abcam, UK, 1:2000), IL-1β (ab205924, Abcam, UK, 1:1000), TNF-α (ab66579, Abcam, UK, 1:1000), IL-6 (MAB406, R&D Systems, Minneapolis, USA, 1:1000), TGF-β1 (ab179695, Abcam, UK, 1:5000), COL1A1 (ab254113, Abcam, UK, 1:1000), and COL3A1 (ab7778, Abcam, UK, 1:1000) and GAPDH (ab8245, Abcam, UK, 1:5000).

### RNA immunoprecipitation

We used a Magna RIP RNA-binding protein immunoprecipitation kit (Millipore, Burlington, MA, USA) to assess the interaction of ZFP36 with CREBBP mRNA 3ʹUTR. Co-immunoprecipitation was performed using antibody toward ZFP36 (#71632, Cell Signaling Technology, USA, 1:1000). RNA was quantified by qRT-PCR.

### Pull down

RNA was first labeled with biotin using a Biotin-RNA Labeling Mix (Roche, Basel, Switzerland) following the manufacturer’s instructions. Cells were sonicated in lysis buffer (20 mM Tris-HCl, pH7.5, 150 mM NaCl, 0.5 mM EDTA, 0.5% NP-40, and a cocktail of protease, phosphatase, and RNase inhibitors) and then centrifuged for 10 min at 13,000 × *g* at 4 °C. The supernatant was collected and incubated with 2 μg biotin-labeled RNA for 2 h at 4 °C. Dynabeads (Invitrogen, Carlsbad, CA, USA) were added to the sample and incubated for a further 1 h at 4 °C. The interacting proteins were isolated according to the manufacturer’s instructions and then subjected to SDS-PAGE.

### Histopathological analysis

Lung tissue was fixed in 10% buffered formalin, embedded in paraffin, and then sectioned into 5 μm slices. The tissue sections were stained with H&E or Masson’s trichrome stain and then assessed by light microscopy. Lung tissue was scored according to severity (0–3, with 3 as severe). The presence of intra-alveolar hemorrhage and debris, cellular hyperplasia, hyperemia, and congestion were considered in the representation of acute lung injury.

### TUNEL assay

Apoptosis was determined by using a TUNEL-based colorimetric apoptosis detection system (Dead End, Promega Corporation, Madison, WI, USA) according to the manufacturer’s instructions. The percentage of TUNEL-positive cells in 20 random fields was used to calculate the rate of apoptosis.

### Immunofluorescence and immunohistochemical staining

After deparaffinization, lung tissue sections were permeabilized with 0.1% Triton X-100 and blocked with bovine serum albumin (5%). Tissue samples were then incubated with primary antibodies overnight at 4 °C. After several washes in phosphate-buffered saline, sections were incubated with green or red-fluorescent Alexa Fluor IgG Ab (Invitrogen). Nuclei were stained with DAPI and then images were obtained using a fluorescence microscope (Olympus, Tokyo, Japan).

### Cell culture and treatment

MLE-2 cells were cultured in RPMI 1640 supplemented with 5% fetal bovine serum at 37 °C and 5% CO_2_. ZFP36 and CREBBP shRNA and expression vectors were obtained from Gene Pharma (Shanghai, China). Vectors and shRNA were transfected into cells using Lipofectamine RNAi MAX (Invitrogen) following the manufacturer’s instructions,. MLE-2 cells were subjected to 2 h of hypoxia by incubation in an anaerobic chamber at 85% N_2_, 5% H_2_, 10% CO_2_, and 35 °C. Cells were removed from the anaerobic chamber and regenerated for 0, 3, 6, and 12 h in a normal incubator in fresh media.

### Analysis of apoptosis by flow cytometry

To determine apoptosis under each experimental condition, cells were double-stained with Annexin V-FITC and propidium iodide for 15 min, and viability was assessed by flow cytometric analysis (BD Biosciences, San Jose, CA, USA).

### Assay of lung tissue cytokines

Frozen lung tissues were homogenized on ice using a homogenizer and centrifuged at 4000 *g* for 10 min at 4 °C. Levels of tumor necrosis factor alpha (TNF-α) and interleukin 6 (IL-6) in the supernatant were assayed according to the manufacturer’s instructions using ELISA kits (R&D Systems, Minneapolis, MN, USA).

### Statistical analysis

All results are expressed as the means ± standard deviation (SD). Statistical analysis was performed using the Student’s *t*-test, and one-way analysis of variance (ANOVA) was used for multiple comparisons. *P*-values <0.05 indicate a statistically significant difference.

## Supplementary information

Supplementary Figure Legends

Figure S1

Figure S2

Figure S3

Figure S4

## Data Availability

The datasets used and/or analyzed during the current study are available from the corresponding author on reasonable request.
