# Supplementary material for: ZFP36 protects lungs from intestinal I/R-induced injury and fibrosis through the CREBBP/p53/p21/Bax pathway
Source: Cell Death Dis. 2021 Jul 8;12(7):685. doi: 10.1038/s41419-021-03950-y (PMC8266850; doi:10.1038/s41419-021-03950-y)
Supplement: Supplementary file 1 — Supplementary Figure Legends [file 41419_2021_3950_MOESM1_ESM.docx]

**Supplementary Materials**

Fig. S1. Intestinal ischemia–reperfusion (I/R)-induced acute lung injury.

Fig. S2. ZFP36 inhibits apoptosis via the CREBBP/p53/p21/Bax pathway in vitro.

Fig. S3. ZFP36 relieves intestinal ischemia–reperfusion (I/R)-induced lung fibrosis via epithelial–mesenchymal transition in vitro.

Fig. S4. Supplementary Materials of Figure.1F and Figure.5B

Supplementary **Figure Legends**:

**Figure S1**. Intestinal ischemia–reperfusion (I/R)-induced acute lung injury. C57BL/6 mice were subjected to 60 min intestinal ischemia followed by 0, 30, 60, and 90 min reperfusion as indicated. Sham mice were included as control. A–C, Arterial blood PaO_2_ (A), lung water content (B), and the protein content of BALF (C) were measured. D, Representative H&E-stained lung sections of each group. Red arrows outline collapsed alveoli; Blue arrows outline multiple inflammatory cells infilitration; Black arrows outline bronchial hemorrhage. Scale bars: 50 μm. E–G, Levels of IL-1β (E), TNF-α (F), and IL-6 (G) were measured by ELISA and western blotting.

**Figure S2**. ZFP36 inhibits apoptosis via CREBBP/p53/p21/Bax pathway in vitro. Mouse lung epithelial (MLE)-2 cells were subjected to 2 h of hypoxia followed by 0, 3, 6, or 12 h of regeneration. A–C, Levels of IL-1β (A), TNF-α (B), IL-6 (C) were measured by ELISA. D-F, The relative mRNA and protein (F) of ZFP36 (D), and CREBBP (E) were measured by RT-PCR and western blotting. G, Western blot analysis of apoptosis-related protein in cells transfected with shZFP36 or scr. H/R group: 2 h of hypoxia followed by 12 h of regeneration. H, Cell apoptosis was determined by flow cytometry with Annexin V-FTIC and PI double staining in each group.

**Figure S3.** ZFP36 relieves intestinal ischemia–reperfusion (I/R)-induced lung fibrosis by EMT in vitro. A–E, Relative mRNA and protein (E) levels of ZFP36 (A), TGF-β1 (B), COL1A1 (C), and COL3A1 (D) were measured by RT-PCR and western blotting. F, Western blot analysis of EMT related protein in cells transfected with shZFP36 or scr. G and H, Immunofluorescence staining of E-cadherin (G) and α-SMA (H) in cells transfected with shZFP36 or scr. Scale bars: 50 μm.

**Figure S4.** Supplementary Materials of Figure.1F and Figure.5B. A, Western blot analysis of IL-1β, TNF-α and IL-6. B, Apoptosis assay with TUNEL staining. Blue: DAPI. Green: TUNEL.
