# Supplementary figures and images for: ZFP36 protects lungs from intestinal I/R-induced injury and fibrosis through the CREBBP/p53/p21/Bax pathway
Source: Cell Death Dis. 2021 Jul 8;12(7):685. doi: 10.1038/s41419-021-03950-y (PMC8266850; doi:10.1038/s41419-021-03950-y)

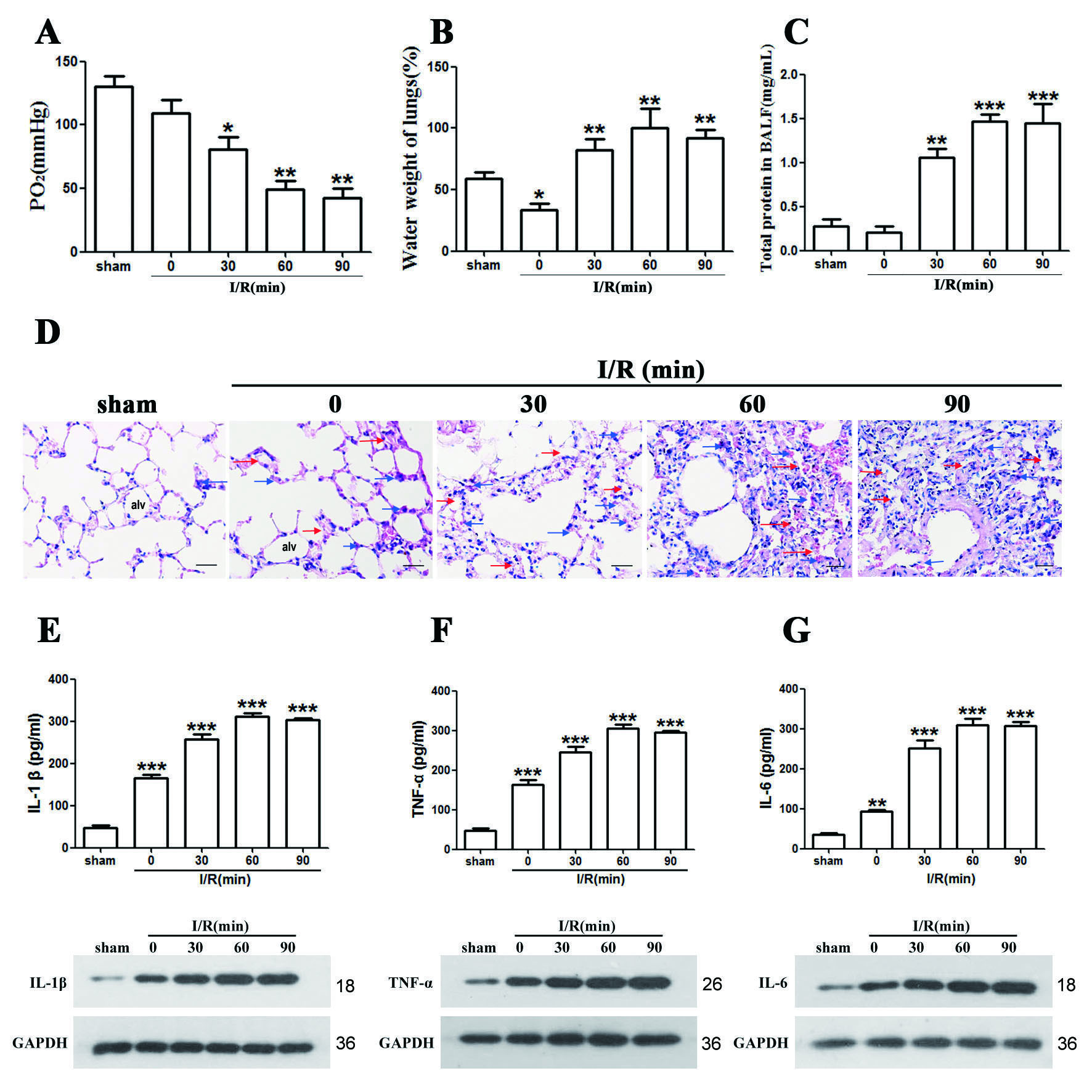

Supplement: Supplementary file 2 — Figure S1 [file 41419_2021_3950_MOESM2_ESM.jpg]

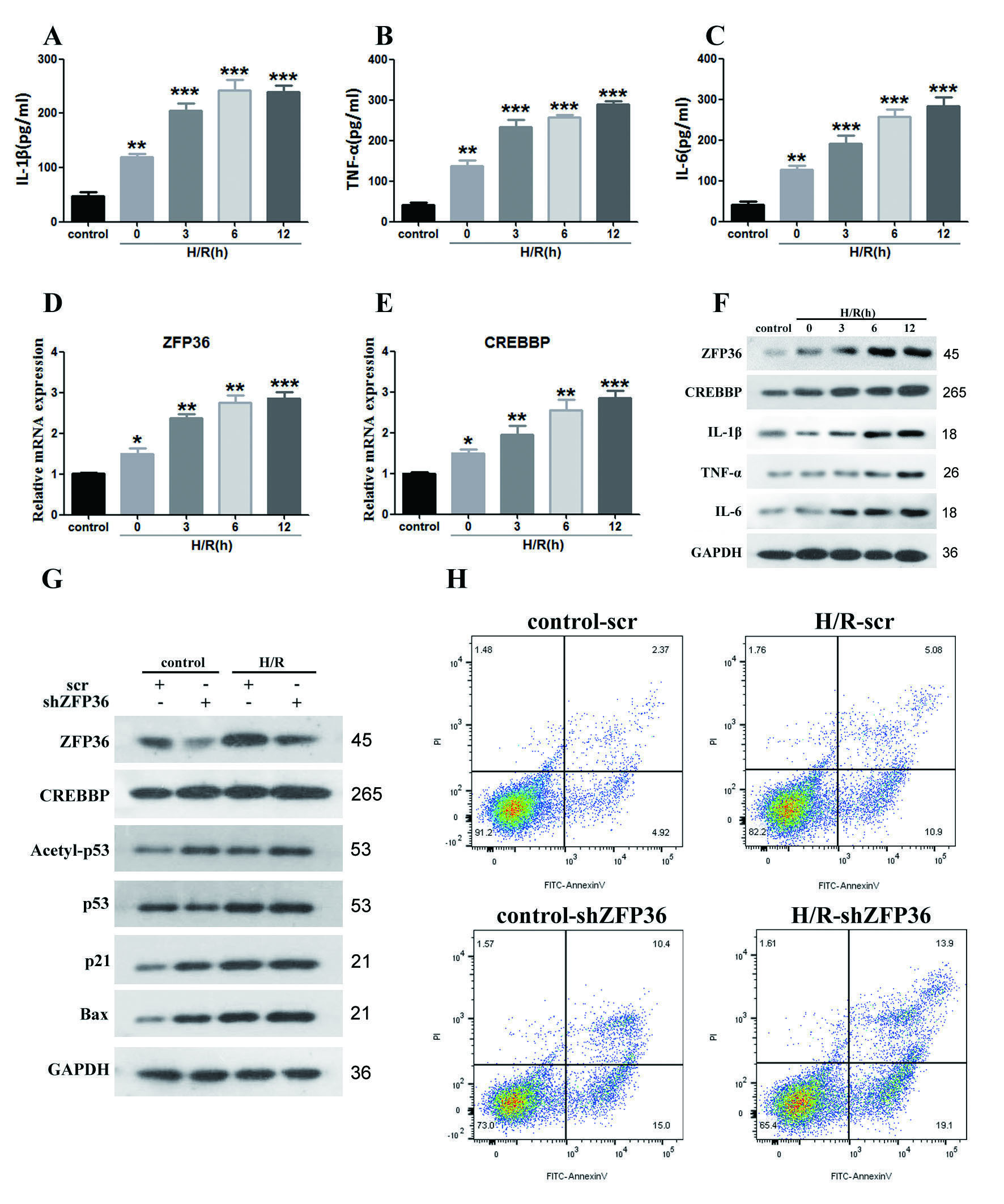

Supplement: Supplementary file 3 — Figure S2 [file 41419_2021_3950_MOESM3_ESM.jpg]

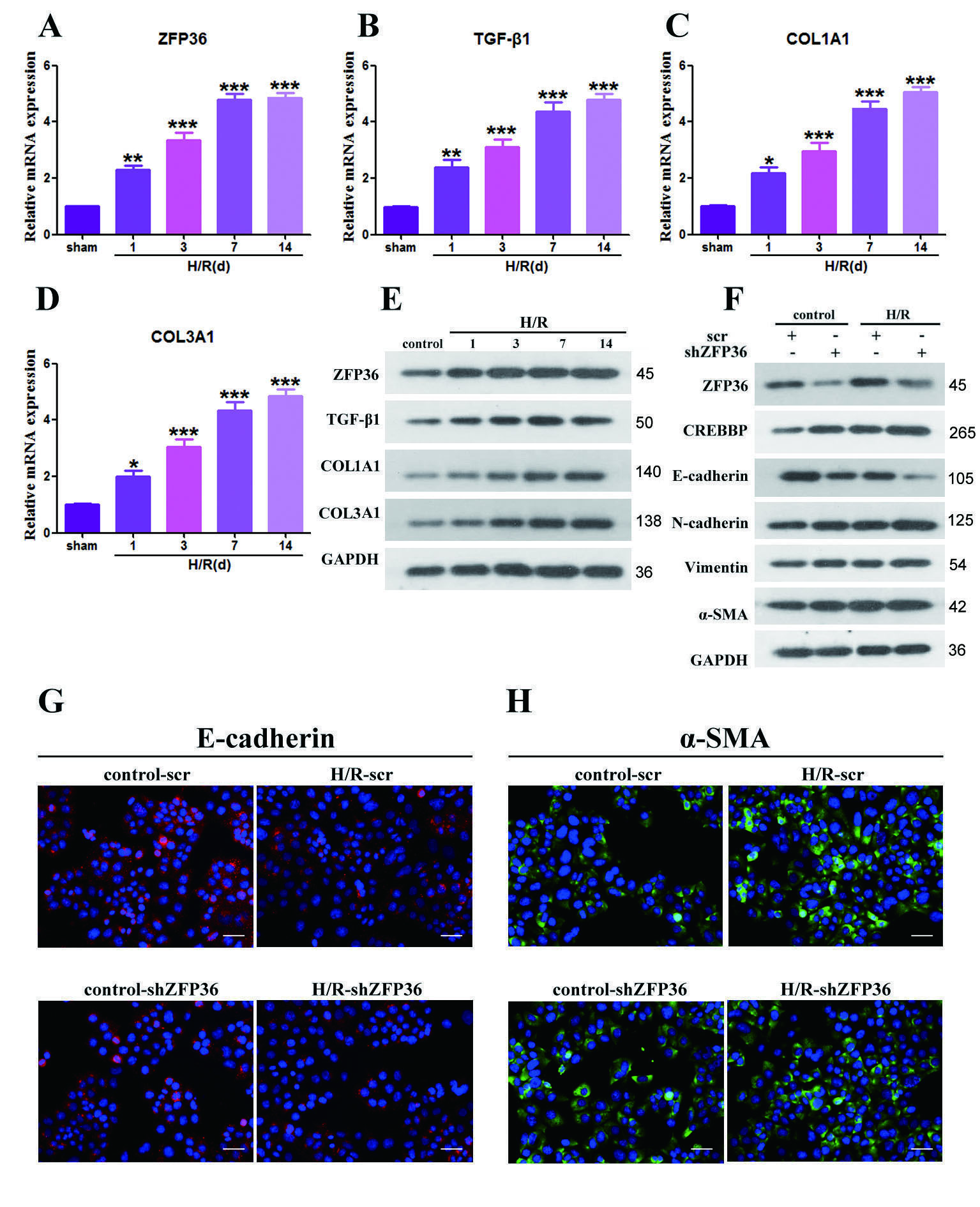

Supplement: Supplementary file 4 — Figure S3 [file 41419_2021_3950_MOESM4_ESM.jpg]

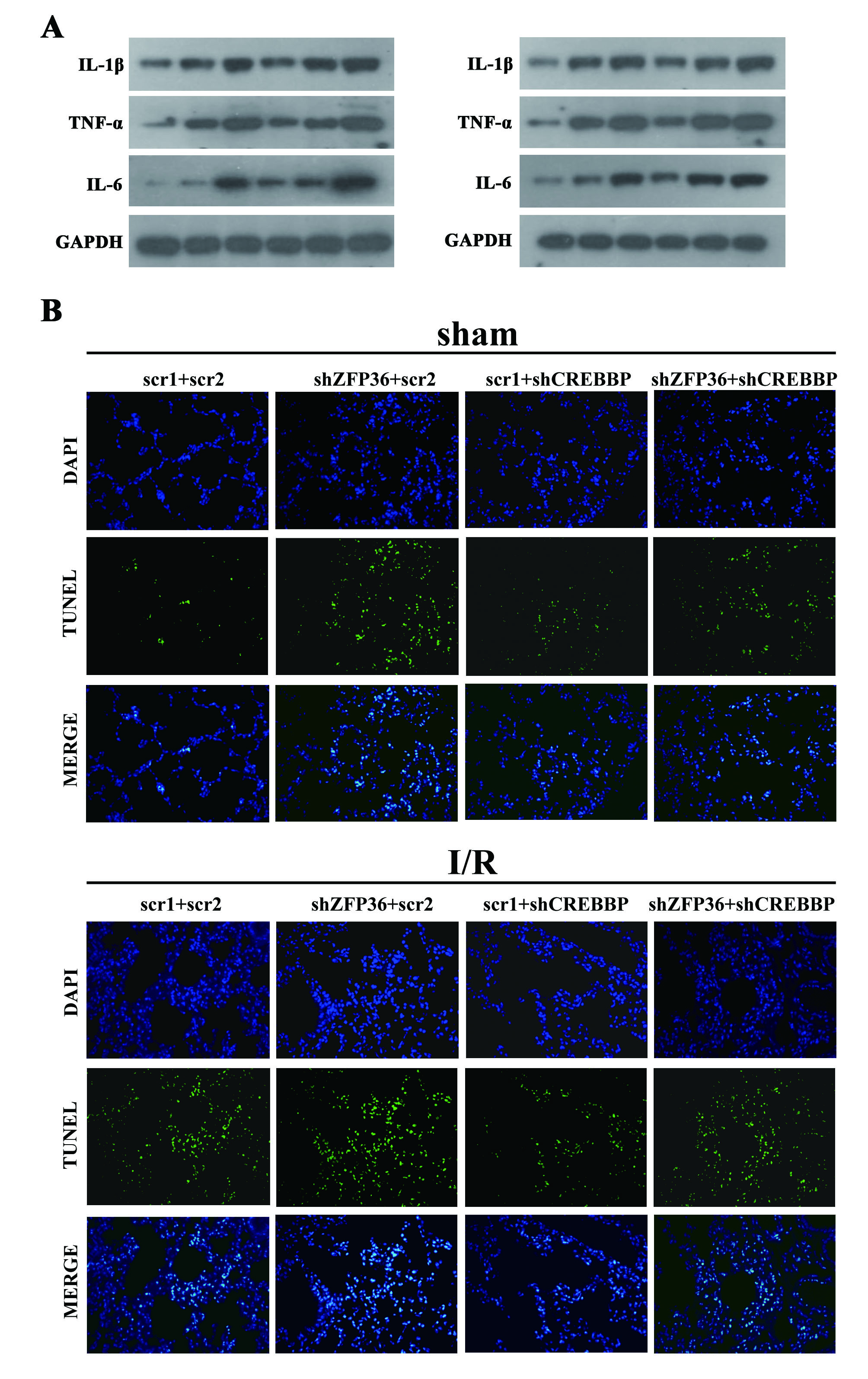

Supplement: Supplementary file 5 — Figure S4 [file 41419_2021_3950_MOESM5_ESM.jpg]
